# 

**Published:** 1877-12

**Authors:** 


### CONTENTS.

### COMMUNICATIONS.

Address ..... 493
Tooth Bearing Tumors of the Jaw ..... 502
Physiology and Treatment of Dental Pulp ..... 507

### SELECTIONS.

Extracts from an article on Popular Dentistry ..... 512
A case of Repeated Excision of the Inferior Dental and Gustatory Nerves, for the Cure of Dental Neuralgia ..... 516
Rest ..... 520
The Dangers of Ether ..... 524

### EDITORIAL.

Boston Journal of Chemistry ..... 525
Virginia State Dental Association ..... 525
What's the Matter with Riggs? ..... 526
Independent Journalism. ..... 528
Stop ..... 530
Close of Volume ..... 531
Index of Diseases and their Treatment ..... 532

